# Endovascular Treatment of Infrapopliteal Arterial Disease in Patients with Diabetic Foot Ulcer: A Systematic Review of Randomised Controlled Trials

**DOI:** 10.1007/s00270-026-04436-0

**Published:** 2026-04-23

**Authors:** Carolina Dutra Queiroz Flumignan, Vinicius Cardoso de Farias, Fabio Cabral Freitas Amaral, Ronald Luiz Gomes Flumignan, Luis Carlos Uta Nakano, José Carlos Costa Baptista-Silva

**Affiliations:** 1 Division of Vascular and Endovascular Surgery, Department of Surgery, Universidade Federal de São Paulo, 754, Rua Borges Lagoa, São Paulo, SP 04038-001 Brazil; 2 Centro Universitário São Camilo, São Paulo, Brazil; 3 Cochrane Brazil, Universidade Federal de São Paulo, São Paulo, Brazil

**Keywords:** Diabetes mellitus, Diabetic foot ulcer, Endovascular revascularisation, Infrapopliteal disease, Percutaneous transluminal angioplasty, Systematic review, Meta-analysis

## Abstract

**Background:**

Diabetes mellitus is strongly associated with peripheral arterial disease and foot ulceration, frequently requiring revascularisation to promote wound healing. Percutaneous transluminal angioplasty (PTA) is widely used for infrapopliteal arterial disease; however, its effectiveness for ulcer healing compared with alternative strategies remains uncertain.

**Objective:**

To assess the effects of PTA in infrapopliteal arterial disease for diabetic ulcer healing.

**Methods:**

We conducted a systematic review of randomised controlled trials identified through MEDLINE, Embase, LILACS, CENTRAL, CINAHL, ClinicalTrials.gov, the World Health Organisation International Clinical Trials Registry Platform, and grey literature sources. Study selection and data extraction were performed independently. Risk of bias was assessed using the Cochrane Risk of Bias tool. Meta-analyses were undertaken where appropriate, and certainty of evidence was evaluated.

**Results:**

Of 34 542 records screened, six randomised controlled trials (945 total participants) were included. Compared with venous bypass, PTA was associated with a higher likelihood of ulcer healing (risk ratio 1.20; 95% confidence interval 1.07–1.33; *p* = .0001; low-certainty evidence). No statistically significant differences were observed between revascularisation strategies for mortality or major amputation. Similarly, no significant differences were identified in comparisons of PTA versus drug-coated balloon or drug-coated stent interventions.

**Conclusion:**

PTA may be associated with improved arterial ulcer healing compared with venous bypass in patients with diabetes, without clear differences in mortality or amputation rates. However, the certainty of evidence is low, and these findings should be interpreted with caution. Further adequately powered randomised trials are required to clarify the comparative effectiveness of infrapopliteal revascularisation strategies.

**Graphical abstract:**

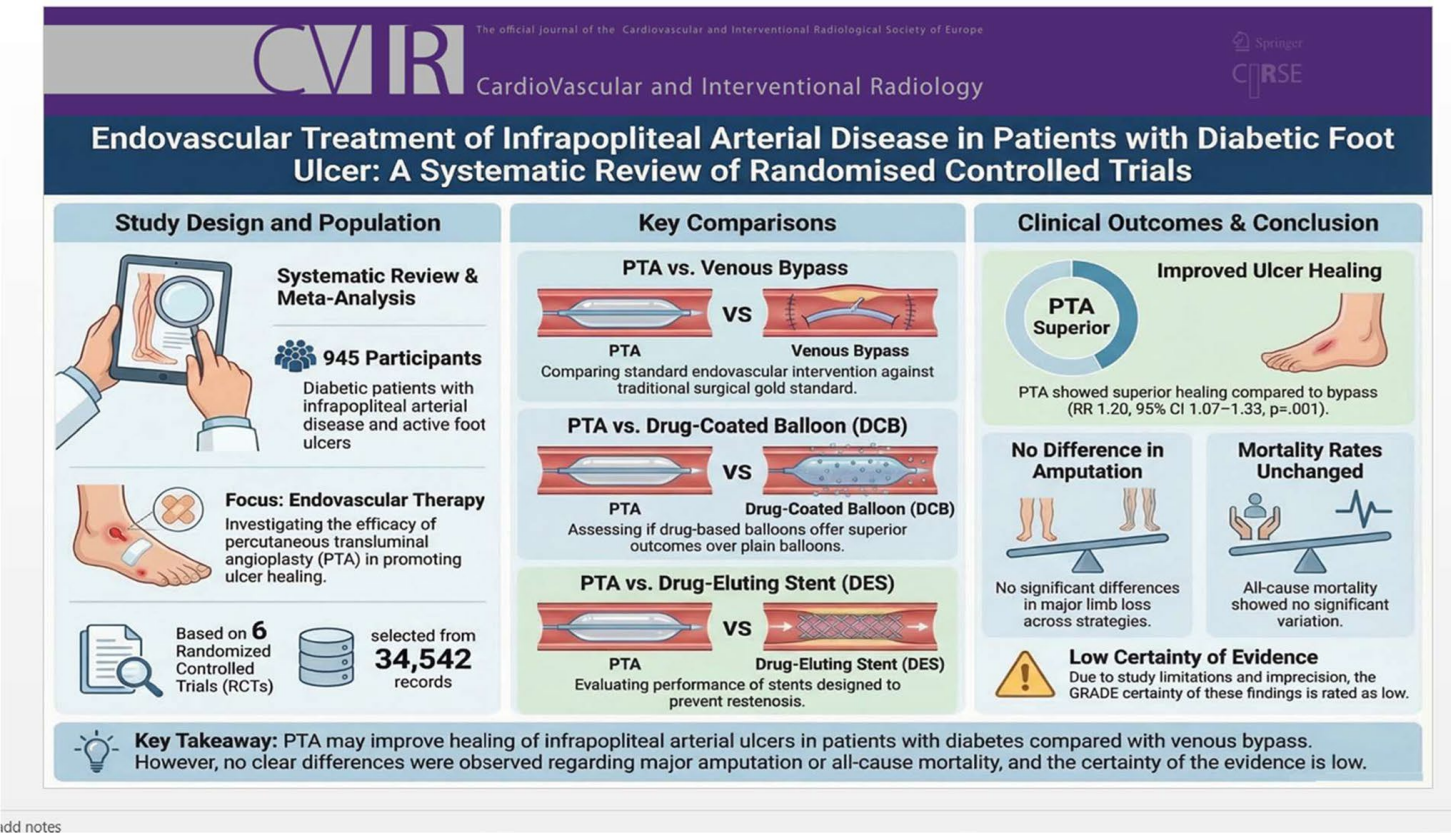

**Supplementary Information:**

The online version contains supplementary material available at 10.1007/s00270-026-04436-0.

## Introduction

Arterial ulcers of the lower limbs represent a severe manifestation of peripheral arterial disease (PAD), particularly among patients with diabetes mellitus [1,2]. The interplay between chronic ischaemia, microvascular dysfunction, and peripheral neuropathy contributes to impaired wound healing and ulcer progression [3,4]. It is estimated that more than 85% of individuals with diabetes will develop PAD during their lifetime, and approximately 10%–25% will develop foot ulcers, with a substantial risk of infection, gangrene, and limb loss.^[[4,5]]^ These complications are associated with marked reductions in quality of life and impose a considerable burden on healthcare systems.

Chronic limb-threatening ischaemia (CLTI), the most advanced stage of PAD, is associated with high rates of morbidity and mortality [6]. In the absence of revascularisation, up to 40% of patients may require major amputation within one year, and annual mortality may exceed 20%[7]. Even following successful revascularisation, many patients undergo repeated interventions and experience frequent hospitalisations owing to recurrent vascular events and coexisting cardiovascular disease.

Management of arterial ulcers in diabetic patients requires a multidisciplinary approach, encompassing glycaemic control, infection management, wound care, and restoration of limb perfusion. Percutaneous transluminal angioplasty (PTA) has become a widely adopted endovascular strategy for infrapopliteal arterial disease, offering high technical success rates and lower peri-operative morbidity compared with surgical venous bypass [8,9]. However, the long-term durability of PTA is limited by restenosis, driven not only by neointimal hyperplasia, but also by elastic recoil and negative remodelling—mechanisms that are particularly relevant in long, calcified lesions, which are characteristic of infrapopliteal arterial disease [10–12].

To address restenosis, drug-based endovascular technologies, including drug-coated balloons (DCB) and drug-eluting stents (DESs), have been developed with the aim of improving vessel patency [13–16]. Nevertheless, their role in infrapopliteal arteries remains controversial, and published trials have reported inconsistent results with respect to ulcer healing, limb salvage, and the need for repeat revascularisation [17,18].

Beyond uncertainties related to device performance, variability in clinical practice and adherence to evidence-based recommendations further complicate decision-making in this population [19–22].

Given the ongoing uncertainty regarding the optimal revascularisation strategy for arterial ulcers in patients with diabetes, a synthesis of high-quality evidence is warranted. This systematic review aims to evaluate the effects of PTA for infrapopliteal arterial disease in diabetic patients, focussing on clinically relevant outcomes including ulcer healing, amputation, mortality, and health-related quality of life, in order to inform evidence-based clinical decision-making.

## Methods

Study selection was performed independently by two authors, who also evaluated the data extracted from the included studies. Risk of bias version 1 (RoB 1) was assessed using the Cochrane risk of bias tool [23]. For studies that allowed, we performed qualitative synthesis and quantitative meta-analysis. Data were analysed using the randomised-effects model when there is more than one included study, with analyses conducted using the RevMan statistical software [24].

### Study Design and Protocol

This study was conducted as a systematic review of randomised controlled trials evaluating the effects of percutaneous transluminal angioplasty (PTA) for the treatment of arterial ulcers of the lower limbs in patients with diabetes mellitus. The methodology followed a previously published and registered protocol [25]. The review was conducted in accordance with the Preferred Reporting Items for Systematic Reviews and Meta-Analyses (PRISMA) statement and the Cochrane Handbook for Systematic Reviews of Interventions [26,27].

### Data Sources and Search Strategy

A comprehensive literature search was undertaken in the following electronic databases: MEDLINE (via PubMed), Embase, LILACS, the Cochrane Central Register of Controlled Trials (CENTRAL), IBECS, CINAHL, AMED, the World Health Organisation International Clinical Trials Registry Platform, ClinicalTrials.gov, and OpenGrey. Searches combined free-text terms and controlled vocabulary (MeSH and Emtree), including but not limited to *foot ulcer*, *leg ulcer*, *diabetic foot*, *peripheral arterial disease*, *diabetes complications*, *peripheral vascular diseases*, *critical limb-threatening ischaemia*, *below-the-knee ulcer*, *angioplasty*, *stents*, and *endovascular procedures*.

No restrictions were applied with regard to language or year of publication. The full search strategy for MEDLINE is provided in Supplementary Appendix 1, and database-specific strategies are available from the authors upon reasonable request.

### Eligibility Criteria

Randomised controlled trials were eligible for inclusion if they enrolled adult patients with type 1 or type 2 diabetes mellitus presenting with arterial ulceration below the knee and objective clinical and imaging evidence of infrapopliteal peripheral arterial disease.

Eligible interventions included PTA of infrapopliteal arteries. Comparators comprised best medical therapy alone (with or without placebo), open surgical revascularisation, or alternative endovascular strategies. Where reported separately, different endovascular technologies (e.g., standard balloon angioplasty, DCB, DESs) were analysed as distinct comparisons.

### Outcomes

The primary outcome was arterial ulcer healing. Secondary outcomes included major amputation, all-cause mortality, adverse events related to revascularisation, and health-related quality of life.

### Study Selection and Data Extraction

Titles and abstracts were independently screened by two reviewers. Full-text articles were retrieved for all potentially eligible studies and assessed independently against the inclusion criteria. Data extraction was performed independently by the same reviewers using Rayyan software and a predefined data extraction form [28]. Disagreements at any stage were resolved by discussion and consensus.

### Risk of Bias Assessment

The risk of bias of included studies was assessed independently by two reviewers using the Cochrane RoB 1 [23]. Domains evaluated included random sequence generation, allocation concealment, blinding, incomplete outcome data, selective reporting, and other sources of bias.

### Data Synthesis and Statistical Analysis

A qualitative synthesis was performed for all included studies. Where sufficient data were available, quantitative meta-analyses were conducted. Effect estimates were pooled using a random-effects model. Results were expressed as risk ratios (RR) with corresponding 95% confidence intervals (CI). Statistical analyses were performed using Review Manager (RevMan) software [24]. The certainty of the evidence for each outcome was assessed using the Grading of Recommendations Assessment, Development and Evaluation (GRADE) approach, considering study limitations, inconsistency, indirectness, imprecision, and publication bias and using the GRADEpro software [29,30].

## Results

The risk of bias assessment revealed heterogeneous methodological quality across the included studies and is reported alongside the corresponding meta-analyses, as shown in related figures. The studies included in the qualitative analysis’ summary are detailed in Table 1. The only study that was not included in quantitative synthesis did not separate the results by person, but by wound [31].

**Table 1:** Characteristics of the studies included in the qualitative synthesis

Study (year)	Study design	Setting	Participants (n)	Intervention	Comparator
Bradbury et al. [32]	Randomised, open-label trial	Multicentre	345	PTA	Surgical venous bypass
Liistro et al. [10]	Randomised, open-label trial	Single centre	158	Paclitaxel drug-eluting balloon angioplasty	Plain balloon PTA
Patel et al. [33]	Randomised, double-blind trial	Two centres	138	Drug-coated balloon angioplasty	Plain balloon PTA
Schulte et al. [34]	Randomised trial	Multicentre	92	Primary stent angioplasty	PTA with bailout stenting
Spreen et al. [35]	Randomised, open-label trial	Multicentre	140	Drug-eluting balloon angioplasty	Plain balloon PTA
Zeller et al. [36]	Randomised, open-label trial	Multicentre	72	Drug-eluting balloon angioplasty	Plain balloon PTA
*Katsanos et al. [31]	Randomised, multicentre trial	Multicentre	~ 200	Sirolimus-eluting stent for infrapopliteal lesions	Plain balloon PTA

^*^ Study included only in the qualitative analysis. PTA: Percutaneous transluminal angioplasty

### Study Selection

The electronic searches identified 34 542 records in April 2020, with a further 6 583 records retrieved during an updated search in January 2025. After removal of duplicates, 36 602 unique records were screened. Of these, 36 584 were excluded at the title and abstract stage as they did not meet the eligibility criteria.

**Fig. 1:**
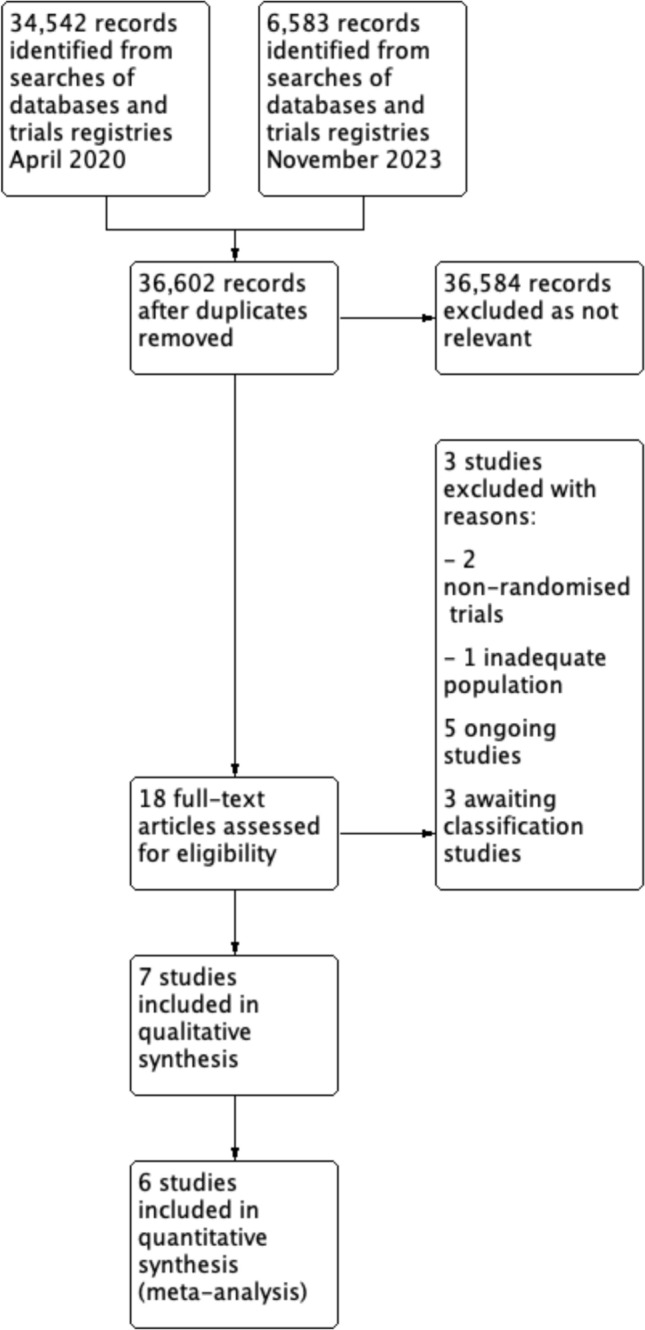
Preferred Reporting Items for Systematic Reviews and Meta-Analyses (PRISMA) flow diagram illustrating the study selection process

Eighteen full-text articles were assessed for eligibility. Eleven studies were excluded for the following reasons: two were non-randomised trials, one included an inappropriate population, five were ongoing studies, and three were classified as awaiting further information. Ultimately, seven randomised controlled trials were included in the qualitative synthesis, and six (945 total participants) were eligible for inclusion in the quantitative synthesis (meta-analysis). The study selection process is summarised in the PRISMA flow diagram (Fig. 1) [10,31–36].

### Study Characteristics

The main characteristics of the studies included in the qualitative synthesis are summarised in Table 1. All included trials evaluated infrapopliteal revascularisation strategies in patients with diabetes and arterial ulceration. One study was excluded from the meta-analysis because outcomes were reported per wound rather than per patient, precluding appropriate pooling of data[31].

### Risk of Bias

The methodological quality of the included trials was variable. The risk of bias assessment demonstrated heterogeneity across studies, particularly in domains related to blinding and outcome assessment. Detailed risk of bias evaluations are presented alongside the corresponding forest plots in the meta-analysis figures.

## Ulcer Healing

**Fig. 2:**
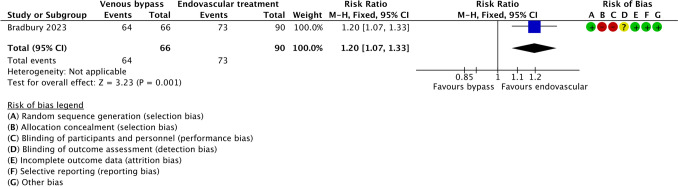
Forest plot comparing ulcer healing between venous bypass and endovascular revascularisation in patients with diabetes and infrapopliteal arterial disease. Effect estimates are presented as risk ratios with 95% confidence intervals

In the study by Bradbury et al. [32], which compared surgical venous bypass with endovascular revascularisation, ulcer healing occurred more frequently in the endovascular group than in the venous bypass group. Healing events were reported in 73 of 90 patients treated with endovascular therapy and in 64 of 66 patients undergoing venous bypass. This corresponded to a pooled risk ratio of 1.20 (95% confidence interval 1.07–1.33; Z = 3.23; *p* = 0.001), indicating a statistically significant effect favouring endovascular treatment (Fig. 2). Despite the observed difference in ulcer healing, the certainty of the evidence was rated as low according to the GRADE approach, primarily owing to study limitations and imprecision (Table 2).

**Table 2:** GRADE assessment of the certainty of evidence comparing open surgical versus endovascular revascularization strategies

Open compared to endovascular treatment for below the knee ulcers in diabetic patients					
Outcomes	No of participants (studies) Follow-up	Certainty of the evidence (GRADE)	Relative effect (95% CI)	Anticipated absolute effects	
				Risk with endovascular treatment	Risk difference with Open
Wound healing	156 (1 RCT)	⨁⨁◯◯ Low^a,b^	**RR 1.20** (1.07 to 1.33)	811 per 1.000	**162 more per 1.000** (from 57 to 268 more)
Amputation Rate	345#(1 RCT)	⨁⨁◯◯ Low^a,b^	**RR 1.10** (0.72 to 1.69)	185 per 1.000	**18 more per 1.000** (from 52 fewer to 128 more)
All cause death	345#(1 RCT)	⨁⨁◯◯ Low^a,b^	**RR 1.19** (0.96 to 1.48)	445 per 1.000	**85 more per 1.000** (from 18 fewer to 214 more)
Vascular QoL questionnaire	136#(1 RCT)	⨁⨁◯◯ Low^a,b^	–	The mean vascular QoL questionnaire with endovascular was **4.6**	MD **0.2 higher** (0.27 lower to 0.67 higher)

^*^The risk in the intervention group (and its 95% confidence interval) is based on the assumed risk in the comparison group and the relative effect of the intervention (and its 95% CI)

GRADE Working Group grades of evidence

High certainty: we are very confident that the true effect lies close to that of the estimate of the effect

Moderate certainty: we are moderately confident in the effect estimate: the true effect is likely to be close to the estimate of the effect, but there is a possibility that it is substantially different

Low certainty: our confidence in the effect estimate is limited: the true effect may be substantially different from the estimate of the effect

Very low certainty: we have very little confidence in the effect estimate: the true effect is likely to be substantially different from the estimate of effect

Explanations

a. Downgraded half level due to study limitations: selection and performance bias

b. Downgraded one level for imprecision: a small number of events and participants

GRADE: Grading of Recommendations, Assessment, Development and Evaluation. CI: confidence interval; MD: mean difference; QoL: Quality of Life; RR: risk ratio

**Fig. 3:**
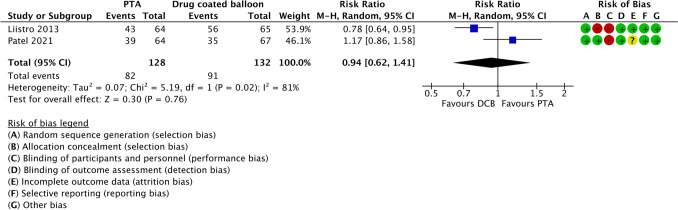
Forest plot comparing ulcer healing between percutaneous transluminal angioplasty and drug-coated balloon angioplasty in patients with diabetes and infrapopliteal arterial disease. Effect estimates are presented as risk ratios with 95% confidence intervals

The comparison of endovascular treatment with percutaneous treatment angioplasty (PTA) with plain balloon versus drug-coated balloon (DCB) was performed with two studies. Liistro[10] and Patel [33] were included, and it was found no significant difference between the two groups (Fig. 3).

### Amputation

For this outcome, three distinct comparisons were identified and are described below.

The study by Bradbury et al.[32] reported no significant difference between the treatment groups, with a RR of 1.10 (95% CI 0.72 to 1.69; Z = 0.43; p = 0.66), indicating that the revascularization strategy did not significantly influence amputation rates (Fig. 7).

**Fig. 4:**
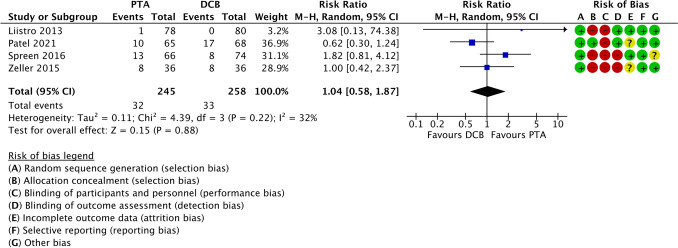
Forest plot comparing amputation rates between percutaneous transluminal angioplasty and drug-coated balloon angioplasty in patients with diabetes and infrapopliteal arterial disease. Effect estimates are presented as risk ratios with 95% confidence intervals. PTA = percutaneous transluminal angioplasty; DCB = drug-coated balloon

Comparisons between endovascular treatment using a plain balloon PTA and DCB were reported by Liistro et al.[10], Spreen et al.[33], Zeller et al.[35], and Patel et al.[36] Pooled analysis demonstrated no significant difference between the techniques (RR 1.04; 95% CI 0.58 to 1.87; *Z* = 0.15; *p* = 0.88), suggesting comparable amputation outcomes between plain balloon PTA and DCB (Fig. 4).

Finally, the comparison between PTA and drug-coated stents was evaluated by Schulte et al.[34], with no statistically significant difference observed between the two approaches, as illustrated in the corresponding forest plot (Fig. 8).

### Mortality

**Fig. 5:**
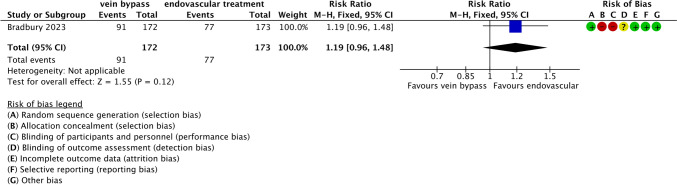
Forest plot comparing all-cause mortality between venous bypass and endovascular revascularisation in patients with diabetes and infrapopliteal arterial disease. Effect estimates are presented as risk ratios with 95% confidence intervals

In the study by Bradbury et al.,[32] mortality did not differ significantly between the venous bypass group (91 events among 172 patients) and the endovascular group (77 events among 173 patients). As this was a single-study comparison, heterogeneity was not applicable (RR 1.19; 95% CI 0.96 to 1.48; Z = 1.55; *p* = 0.12) (Fig. 5).

**Fig. 6:**
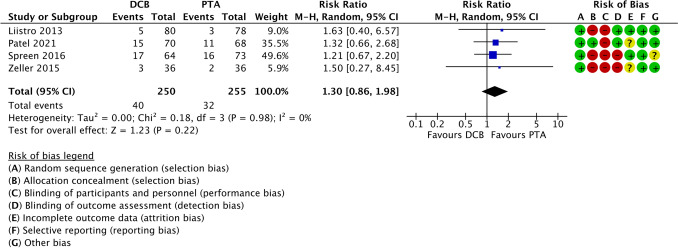
Forest plot comparing all-cause mortality between percutaneous transluminal angioplasty and drug-coated balloon angioplasty in patients with diabetes and infrapopliteal arterial disease. Effect estimates are presented as risk ratios with 95% confidence intervals. PTA = percutaneous transluminal angioplasty; DCB = drug-coated balloon

**Fig. 7:**
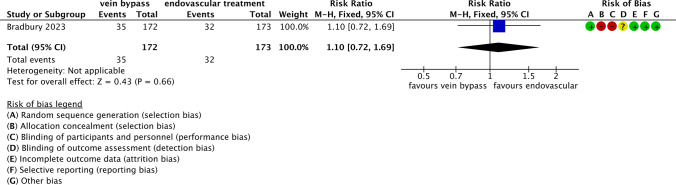
Forest plot comparing amputation rates between percutaneous transluminal angioplasty and vein bypass in patients with diabetes and infrapopliteal arterial disease. Effect estimates are presented as risk ratios with 95% confidence intervals

**Fig. 8:**
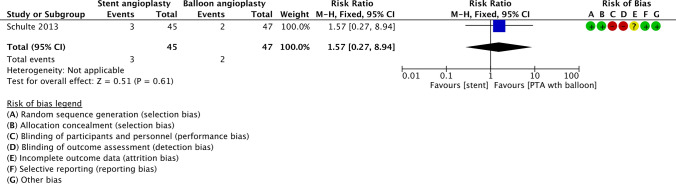
Forest plot comparing amputation rates between percutaneous transluminal angioplasty and drug-coated stents in patients with diabetes and infrapopliteal arterial disease. Effect estimates are presented as risk ratios with 95% confidence intervals

Across the included studies [10,33,35,36] comparing endovascular treatment using plain balloon PTA with DCB, no significant difference in mortality was observed (RR 1.30; 95% CI 0.86 to 1.98). Between-study heterogeneity was low (I2 = 0%), and the overall effect was not statistically significant (*Z* = 1.23; *p* = 0.22), as shown in Fig. 6.

### Quality of Life

**Fig. 9:**
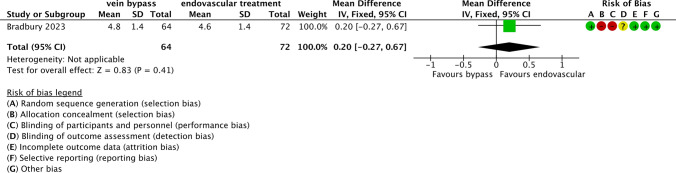
Forest plot comparing vascular quality of life between open surgical revascularisation and endovascular treatment in patients with diabetes and infrapopliteal arterial disease. Effect estimates are presented as mean differences (MDs) with 95% confidence intervals. MD = mean difference

Only Bradbury et al.[32] evaluated quality of life, comparing venous bypass with endovascular treatment. Mean scores were 4.8 (SD 1.4; *n* = 64) in the venous bypass group and 4.6 (SD 1.4; *n* = 72) in the endovascular group. The mean difference was 0.20 (95% CI − 0.27 to 0.67; *Z* = 0.83; *p* = 0.41), indicating no statistically significant difference between groups. As this was a single-study comparison, heterogeneity was not applicable. The risk of bias assessment identified concerns related to selection and performance bias (Table 2; Fig. 9).

## Discussion

Percutaneous transluminal angioplasty (PTA) of infrapopliteal arteries, particularly when combined with drug-based technologies such as DCBs and DESs, has emerged as an attractive strategy for the treatment of arterial ulcers in patients with diabetes and peripheral arterial disease (PAD) [37]. The present systematic review suggests that while endovascular revascularisation is associated with improved ulcer healing, it does not confer a clear advantage over surgical venous bypass in terms of major clinical outcomes such as amputation or mortality. These findings are broadly consistent with previous reports [38].

Drug-eluting balloon technology represents an important innovation in endovascular therapy, with robust evidence demonstrating reduced restenosis and lower rates of target lesion revascularisation (TLR) in femoropopliteal disease [14–16]. However, evidence supporting its effectiveness in infrapopliteal arteries remains inconsistent. The DEBATE-BTK trial by Liistro et al. [10] reported significant reductions in restenosis and TLR at 12 months among diabetic patients with critical limb-threatening ischaemia, without signals of increased mortality, major amputation, or target vessel thrombosis. In contrast, the IN.PACT DEEP trial reported higher rates of major amputation in the DCB-treated group, raising concerns regarding potential distal embolisation of paclitaxel particles and highlighting the importance of meticulous wound surveillance and standardised limb care protocols in this population [36].

Further uncertainty is reflected in the SINGA-PACLI trial conducted by Patel et al.[33], which did not demonstrate superiority of DCB angioplasty over conventional PTA in terms of primary patency and reported lower amputation-free survival in the DCB group. These divergent findings likely reflect the complexity of the infrapopliteal disease phenotype, characterised by diffuse calcification, small vessel calibre, high prevalence of diabetes and renal impairment, and advanced tissue loss [13,39]. Although DCBs may reduce restenosis and TLR, their long-term clinical benefit in this context may be attenuated by disease severity and competing risks.

In the present analysis, no statistically significant differences were observed between PTA (including DCB use) and venous bypass for major outcomes such as amputation or all-cause mortality. This underscores the difficulty of demonstrating superiority of one revascularisation strategy over another when patient-centred outcomes are considered. Although previous analyses have raised concerns regarding increased late mortality associated with paclitaxel-coated devices, particularly in femoropopliteal interventions, [40,41] our pooled estimates did not demonstrate a statistically significant increase in mortality with DCB use (RR 1.30, 95% CI 0.86–1.98). These findings should be interpreted cautiously, given the limited number of trials and the restricted diabetic infrapopliteal population represented in the available data.

Ulcer healing was the only outcome for which endovascular treatment demonstrated a statistically significant benefit, with a pooled risk ratio of 1.20 (95% CI 1.07–1.33). However, this estimate was driven by a single randomised trial, and the certainty of evidence was rated as low. The observed benefit may reflect the capacity of endovascular revascularisation to rapidly restore tissue perfusion and support wound healing, even if long-term durability is compromised by restenosis, as suggested by studies such as DEBATE-BTK and IN.PACT DEEP [10,36].

Interpretation of these findings must account for important methodological limitations, including lack of blinding, potential inadequacies in randomisation, limited adjustment for key clinical confounders (such as glycaemic control, infection status, and wound care), and heterogeneity in lesion characteristics and follow-up protocols. Consistent with these limitations, application of the GRADE approach indicated low certainty of evidence for most outcomes, suggesting that the true effect may differ substantially from the observed estimates. In this context, concerns raised by Katsanos et al. regarding potential late mortality associated with paclitaxel-based devices remain relevant [40], although no statistically significant signal was detected in the present review.

Anatomical characteristics deserve specific attention when interpreting comparative effectiveness across revascularisation strategies. Surgical bypass was more likely to have been performed in patients with adequate distal run-off permitting anastomosis, whereas angioplasty could be applied even in patients with poor distal run-off in the foot [9,32]. Furthermore, the presence of medial arterial calcification and small artery disease is likely to influence outcomes and may differentially affect the performance of endovascular versus surgical approaches [39]. These anatomical factors are rarely captured adequately in available randomised data, limiting cross-study comparisons [32,39]. The confounding influence of underlying comorbidities—including glycaemic control, end-stage renal disease, wound infection, peripheral neuropathy, and microvascular disease—on outcomes such as wound healing also cannot be overlooked, nor can the differential impact of direct versus indirect revascularisation strategies [4,39]. A comprehensive subgroup analysis or multivariate regression to address these factors would ideally enhance the robustness of this review’s findings, but was precluded by limited data availability across the included trials [31–36]. It is also important to recognise that RCTs investigating novel modalities such as DCBs or DESs are conducted under strictly controlled conditions with respect to lesion length and other anatomical factors. Consequently, they may not accurately represent real-world scenarios compared with studies that compare PTA with open surgery [32]. For example, DES trials generally include subjects with limited lesion lengths and preserved distal run-off, which can limit their generalisability to a broader population with more complex disease. [34,36]. Such limitations must also be considered when interpreting the present findings.

Overall, these findings support PTA as a viable revascularisation strategy for infrapopliteal arterial disease in diabetic patients with arterial ulcers, particularly in terms of ulcer healing. However, when more severe outcomes such as amputation-free survival and mortality are considered, no single strategy—whether PTA, PTA with DCB, or venous bypass—can be universally recommended. Treatment decisions should therefore be individualised, taking into account patient comorbidities, anatomical complexity, wound characteristics, and local expertise. Future adequately powered randomised trials with standardised wound surveillance, rigorous follow-up, and patient-centred outcomes are required to clarify the long-term role of DCBs in infrapopliteal revascularisation and their true impact on limb preservation and survival.

## Conclusions

Percutaneous transluminal angioplasty (PTA) may improve healing of infrapopliteal arterial ulcers in patients with diabetes compared with venous bypass. However, no clear differences were observed between revascularisation strategies regarding major amputation or all-cause mortality, and the certainty of the evidence is low.

These findings suggest that while PTA may facilitate tissue perfusion and ulcer healing, this benefit does not necessarily translate into reductions in more severe clinical outcomes. Consequently, revascularisation strategies should be individualised, taking into account patient comorbidities, anatomical complexity, and local expertise.

Further adequately powered randomised controlled trials with standardised wound surveillance and longer follow-up are required to confirm these findings and to better define the role of endovascular therapy in the management of diabetic infrapopliteal arterial disease.

## Clinical Perspective

### What is New?

Percutaneous transluminal angioplasty may be associated with higher rates of arterial ulcer healing than surgical venous bypass in patients with diabetes. However, the certainty of this evidence is low owing to methodological limitations and small sample sizes in the available randomised trials.

### What are the Clinical Implications?

Current evidence is insufficient to support definitive recommendations regarding infrapopliteal revascularisation strategies in diabetic patients. Well-designed, adequately powered randomised controlled trials focussing on patient-centred outcomes, including ulcer healing, limb salvage, and survival, are required to guide clinical decision-making in this growing patient population.

## Electronic supplementary material

Below is the link to the electronic supplementary material.

Supplementary file1 (DOCX 8 KB)

## Appendix

See Figs. 7, 8, 9.
