# Supplementary material for: Endovascular Treatment of Infrapopliteal Arterial Disease in Patients with Diabetic Foot Ulcer: A Systematic Review of Randomised Controlled Trials
Source: Cardiovasc Intervent Radiol. 2026 Apr 23;49(6):1067–78. doi: 10.1007/s00270-026-04436-0 (PMC13212414; doi:10.1007/s00270-026-04436-0)
Supplement: Supplementary file 1 — Supplementary file1 (DOCX 8 KB) [file 270_2026_4436_MOESM1_ESM.docx]

Appendix 1

MEDLINE (via PubMed) search strategy

| 1 Leg Ulcer[mh] 25,99  2 Foot Ulcer[mh] 13,016  3 Leg Ulcer*[tiab] OR Foot Ulcer*[tiab] OR (Plantar Ulcer*[tiab]) OR (arterial ulcer*[tiab]) OR (lower limb ulcer*[tiab]) OR (ulcer* near3 foot[tiab]) OR (ulcer* near3 leg[tiab]) OR below knee ulcer[tiab] 17,251  4 Peripheral Arterial Disease[mh] 19,036  5 Chronic Limb-Threatening Ischemia[mh] 425  6 (Arterial Disease* Peripheral[tiab]) OR (Peripheral Artery Disease*[tiab]) OR (Chronic Limb Threatening Ischemia[tiab]) OR (Critical Limb Ischemia[tiab]) 12,856  7 #1 OR #2 OR #3 OR #4 OR #5 OR #6 56,215  8 Diabetes Mellitus[mh] 513,297  9 Diabetes Insipidus[mh] 8,398  10 Diabetes Complications[mh] 150,725  11 (Diabetes Complication[tiab]) OR (Diabetes-Related Complication*[tiab]) OR (Diabetes Related Complication*[tiab]) OR (Diabetic Complication*[tiab]) OR (Complication* of Diabetes Mellitus[tiab]) OR (Diabetes Mellitus Complication*[tiab]) OR (Diabetes Insipidus[tiab]) OR (Glucose Intolerance[tiab]) OR (Diabetic Foot[tiab]) OR (Diabetic Feet[tiab]) OR (Foot Ulcer Diabetic[tiab]) OR (ulcer* near3 diabetic[tiab]) OR (ulcer* near3 diabetes[tiab]) OR DFU[tiab] 49,734  12 #8 or #9 or #10 or #11 538,792  13 #7 and #12 17,13  14 Angioplasty[mh] 64,53  15 Endovascular Procedures[mh] 147,438  16 Blood Vessel Prosthesis[mh] 50,002  17 Blood Vessel Prosthesis Implantation[mh] 28,025  18 Stents[mh] 90,481  19 angioplas* or percutan* or PTA or recanali* or revascular* or dilat* or endovascular or Intracoil or Zilver PTX or (scoring balloon) or (bare-metal stent*) or (drug-eluting stent*) or (drug-coated balloon) or Supera or Stellarex or AngioSculpt or wolverine or (cutting balloon) or NSE alpha or scoreflex or blimp or "high pressure balloon"[tiab:~4] or bioresorbable scaffolds or "paclitaxel balloon"[tiab:~3] or "sirolimus balloon"[tiab:~3] or "laser intravascular"[tiab:~3] or Atherectomy or "Lithotripsy intravascular"[tiab:~3] 566,8  20 #14 or #15 or #16 or #17 or #18 or #19 644,646  21 randomized controlled trial [pt] 603,656  22 controlled clinical trial [pt] 694,185  23 randomized [tiab] 680,07  24 placebo [tiab] 249,705  25 drug therapy [sh] 2,634,568  26 randomly [tiab] 420,655  27 trial [tiab] 788,79  28 groups [tiab] 2,622,925  29 #21 OR #22 OR #23 OR #24 OR #25 OR #26 OR #27 OR #28 5,873,150  30 animals [mh] NOT humans [mh] 5,165,591  31 #29 NOT #30 5,136,109  32 #13 AND #20 1,636  33 #31 AND #32 464 |
| --- |
